# Impact of Column Support Stiffness on the Mechanical Performance of Flat Frame Structural Systems Supporting Thin-Walled Folded Roofs

**DOI:** 10.3390/ma18010067

**Published:** 2024-12-27

**Authors:** Jacek Abramczyk, Katarzyna Chrzanowska

**Affiliations:** 1 Department of Architectural Design and Engineering Graphics, Rzeszow University of Technology, Al. Powstańców Warszawy 12, 35-959 Rzeszów, Poland; 2 Doctoral School of the Rzeszow University of Technology, Rzeszow University of Technology, Al. Powstańców Warszawy 12, 35-959 Rzeszów, Poland; d565@stud.prz.edu.pl

**Keywords:** flat rod steel frames, structural systems, computer simulations, strength and stability, stiff and semi-rigid joints, thin-walled flat sheets, folded steel roofs, buildings

## Abstract

This article presents a new parametric method for shaping flat transverse frame structural systems supporting thin-walled roofs made of flat sheets folded unidirectionally and transformed elastically to various shell forms. The parameterization was limited to one independent variable, that is the stiffness of the support joints. For different discrete values of simulated stiffness, the surface areas of the cross sections of the tensile and compressed elements and the section modulus of the bending elements were calculated so as to obtain the optimized work of the frame and its elements in the assumed load environment. The developed method allows for optimizing the work of frames considered as flat bar structural systems of building halls, taking into account the ultimate and serviceability limit states. The operation of the method is illustrated with an example concerning the formation of a flat frame working under a load characteristic for buildings located in a lowland area in a moderate climate. The authors intend to successively extend the method with new types of frame systems so as to obtain increasingly accurate and universal models defined by means of an increasing number of independent variables. These parameters are related to different forms and inclinations of columns and girders, and different external load types. The successive increase in the parameters defining the computational parametric model of the frame requires the use of increasingly advanced artificial intelligence algorithms to describe the static and strength performance of the buildings shaped, which makes the proposed method universal and the created structural systems effective in various external environments.

## 1. Introduction

The type of joints used in the designed building structures determines the time and costs of their execution on the one hand. On the other hand, it has a very significant impact on the static and strength performance of these structures, and subsequently on the stability and stress state of both the entire structure and its individual elements, such as columns, purlins and girders [1].

The use of computer technology, especially the FEM method algorithms [2], allows for a very precise analysis of the static and strength work of complex building structural systems, taking into account almost any type of joints: hinged, flexible and rigid. Computer simulations and appropriately adopted simplified static and load schemes lead to a relatively simple analysis of the strength, stability and stiffness of structures, deflections and deformations of their elements and displacements of joints depending on their type [3].

The flat transverse structural systems are most often evenly distributed along the length of the building and stiffened in transverse directions by appropriate wall and roof one- and two-dimensional elements, including thin-walled beam and plates, which allows them to be considered as loaded in their planes [4]. Simulation of the static and strength work of such systems is simplified, and the performed analysis requires a smaller number of independent and dependent variables, as well as limiting optimization conditions.

**Figure 1:**
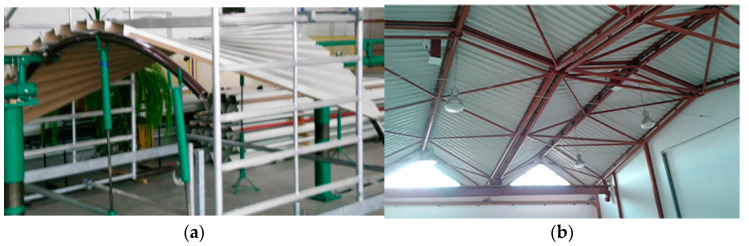
Directrices defining the transformations of roof coverings made of thin-walled trapezoidal sheets nominally flat and corrugated in one direction: (**a**) experimental tests; (**b**) the roof of the laboratory hall used in the previous tests (our own photos).

In the case of roofs made of corrugated sheets without purlins, the lower flanges of the corrugations are supported on roof directrices, Figure 1a,b, which causes the external load to be treated as evenly distributed along the length of the roof directrix, being most often the top girder chord [5]. To limit the vertical displacements of roofs and their structures working under external load, lattice girders and trusses are used, especially for medium-span buildings [6].

To limit the horizontal displacements, rigid supports of columns and connections of both upper and lower chords with columns are used [7]. Changing the joints of individual columns with a foundation from rigid to flexible or hinged causes a significant change in the stiffness of the whole frame system [8]. Therefore, detailed analyses of the work of flat frames with semi-rigid joints are undertaken to an increasingly large extent.

## 2. Critical Review of the Present Knowledge

The thin-walled and unidirectionally corrugated sheets require support in the form of roof directrices passing transversely to the directions of their folds. If a folded roof shell and thus its covering are to be flat, the directrices are rectilinear and most often mutually parallel. In the case when it is advisable to perform a form transformation of the flat folded sheets, the directrices are shaped as mutually oblique curves or straight lines, depending on the type of the surface to which the roof sheeting is curved. Abramczyk presents in the monograph [5] the possibilities of shaping diverse ruled forms of such shells. In this work, it is indicated that the low transverse and torsional stiffness of these sheets and an appropriate assembly technique affect the variety of types of the sheeting designed and the possibility of creating complex structures of many such corrugated shells [9].

In turn, Reichhart designed and carried out an implementation of thin-walled corrugated roofs in the shape of a straight conoid, straight cylindroid and straight hyperbolic paraboloid [10]. He showed that the practical material limitation for the transformation of flat sheets into shells is the maximum unit twist angle of a single transformed fold of about 5 degrees per 1 m along the fold longitudinal axis. Exceeding the limit value by a corrugation working in a shell may result in (1) loss of local stability of the corrugation walls, (2) the appearance of plastic deformations in the areas of wall edges and at the transverse corrugation edges or (3) the impossibility of connecting a new sheet to the one previously fixed in the roof piece due to very large bends and displacements of its thin-walled walls caused by the shape transformation. Because of the need to support the lower flanges of all variously inclined and twisted folds in the roof shell, he used steel girder top chords as directrices, to which the specific tables supporting these flanges could be welded in a relatively easy way [11]. Only this method of steel support is also considered in this article.

The flat frame systems with horizontal directrices allow for the formation of flat roof sheeting only. To force the shape transformations of sheets spread on roof directrices, the transverse flat frame girders have to be inclined to the horizontal [12,13]. Due to the low transverse and torsional stiffness, thin-walled folds can be easily arranged on the directrices within the permissible range of twist of these folds. The angle of twist of each of the folds results from the angle of mutual inclination of the adjacent frame girders and the curvature of the roof shell contraction [5]. Therefore, the mutual position, i.e., the inclination and distance of the adjacent girders (directrices), determines the diversified twist of each shell fold, and therefore its initial stress state.

Therefore, the method of shaping the thin-walled transformed shell forms must take into account the twist and bending of each variously deformed fold and its initial effort and specific work in the roof sheeting [14]. In addition, the folds transfer the load from the roof to the frame girders at the supporting points of their lower flanges that are located at small distances. This allows the load to be treated as evenly distributed along the length of the directrices. Due to these loads occurring along the length of the upper chord and causing its bending, the rod frame girder is not a truss.

**Figure 2:**
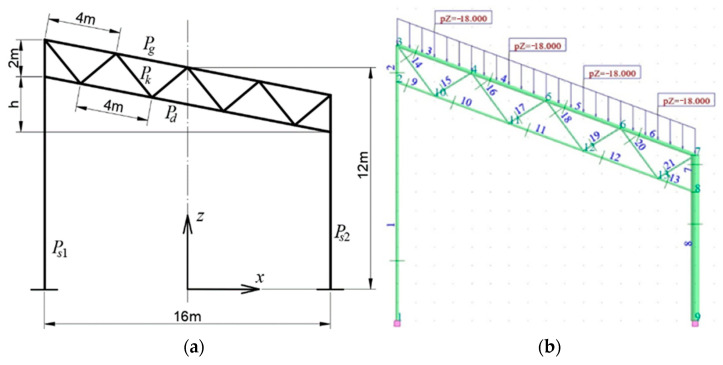
Diagrams of a rectangular trapezoidal configuration with inclined girder: (**a**) geometric characteristics; (**b**) one of load instance (our own photos).

To increase the visual attractiveness of the form of an entire shed or building, vertically inclined columns can be used. Abramczyk and Chrzanowska have analyzed the frame systems of sheds with both inclined girders [15], Figure 2, and inclined columns [16]. They showed that the inclination of each shed roof or wall element has an unfavorable effect on the work of its elements, both in terms of its effort and stability.

In the case of columns, the optimized cross-sections of the lower columns had to be increased to obtain the value of the section module many times higher than in the case of the rectangular frames, for girder inclinations of up to 0.25 [15]. The girder inclination also adversely affects the stability of optimized rectangular trapezial frames, causing a decrease in the static load factor from 7.1 to 5.8 compared to rectangular frames with optimized cross-sections of all elements.

**Figure 3:**
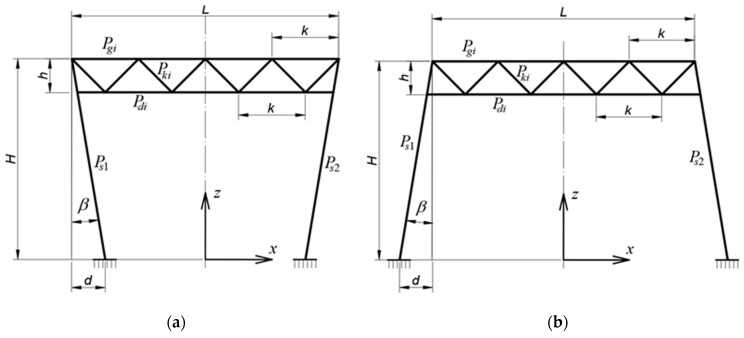
Geometric characteristics of the configurations with inclined columns: (**a**) an inverted trapezial configuration, (**b**) a trapezoidal configuration (our own photos).

The column inclination has an unfavorable effect on the stresses of frame elements, mainly columns, and a positive effect on the overall stability of frames. The inclination of columns is more favorable in inverted trapezoidal systems, Figure 3. The authors have demonstrated the significance of the angles of girder and column inclination on the strength and stability performance of frames treated as flat rod frame structural systems.

In the case of optimized columns, their cross-sections had to be increased to obtain a section module that was twice as high for trapezoidal frames and almost three times as high for inverted trapezoidal frames compared to rectangular frames for girder inclinations of up to 0.33 [16]. Thus, the girder inclination has a negative effect on the strength performance of the examined frames, increasing the strength of some of its elements, including primarily the columns. On the other hand, increasing the column inclination to 0.33 has a positive effect on the overall stability of the frames, causing the critical load factor to increase from 1 to 4 for inverted trapezoidal frames and to 6 for trapezoidal frames compared to rectangular frames with the optimized cross-sections of all elements.

The above analysis shows that it is possible to use the inclinations of the girder and columns as parameters helpful in shaping various types of parametric frames. Both the forms of the entire frame system and their individual elements, including their rectilinearity or curvilinearity, can be subject to geometric parameterization. The diversity of structural system forms is presented by Abdel and Mungan [17], where the form affects the effectiveness of the mechanical performance of the structures. Most of these forms, after a slight modification, can be used as structures of sheds and buildings covered with thin-walled transformed folded sheets. An analysis of the usefulness of structural systems in the design of buildings was carried out by Tarczewski [18].

Mechanical parameterization can be applied to the methods of connecting rod members using joints with different stiffness [19]. This instance is also analyzed in this article. The parameterization can also take into account the stiffness of members, including beams, girders and columns, which influences the mechanical behavior of joints. The construction of rigid or hinged joints working in metal structures is relatively costly and time-consuming. Therefore, semi-rigid ones are currently most commonly used [7,20,21]. Such connections can be easily made of open or closed sections with flat walls [22,23]. The methods of calculating structures with different types of joints are examined by Gomes et al. [24] and Wardenier et al. [25].

Increasingly universal and accurate methods of calculating such structures are being developed, using parametric models [26]. The methods offer calculations related to the effort and stability of the structures [27] and the buckling and warping of their elements [28]. The methods are based on laboratory tests [29] and computer simulations [30,31]. Methods of prediction and optimization of rod lattice structures and steel frames are presented by Deng et al. [32] and Kaveh and Gerami [33]. In defining models used in calculations, artificial intelligence algorithms [34] play an increasingly important role. Artificial shallow and deep neural networks [35,36] allow for the simulation of multi-parameter models helpful in obtaining the appropriate accuracy and short time of calculations. Deep neural networks also play an important role in visual control with various industry specimens measuring the correctness of the performance of the structures and their elements [37].

To optimize the work of the frame structures, genetic algorithms [38] are used. They are based on multi-parameter computational models and search for optimal discrete solutions for the optimization conditions assumed at the beginning of the design process [39]. The authors used this type of algorithm in search of optimal parametric result models working effectively under given load conditions.

## 3. The Aim of the Research

The purpose of the article is to present a parametric mechanical frame model (1) based on the analytical description of the relationship observed during the research and (2) allowing the use (in the process of automation of calculations) of discrete values of parameters defining the designed individual frame flat structural system. Thus, a single-parameter frame model has been developed in which the stiffness (flexibility) of support joints can be adopted in a very wide range of variability.

## 4. Methodology

**Figure 4:**
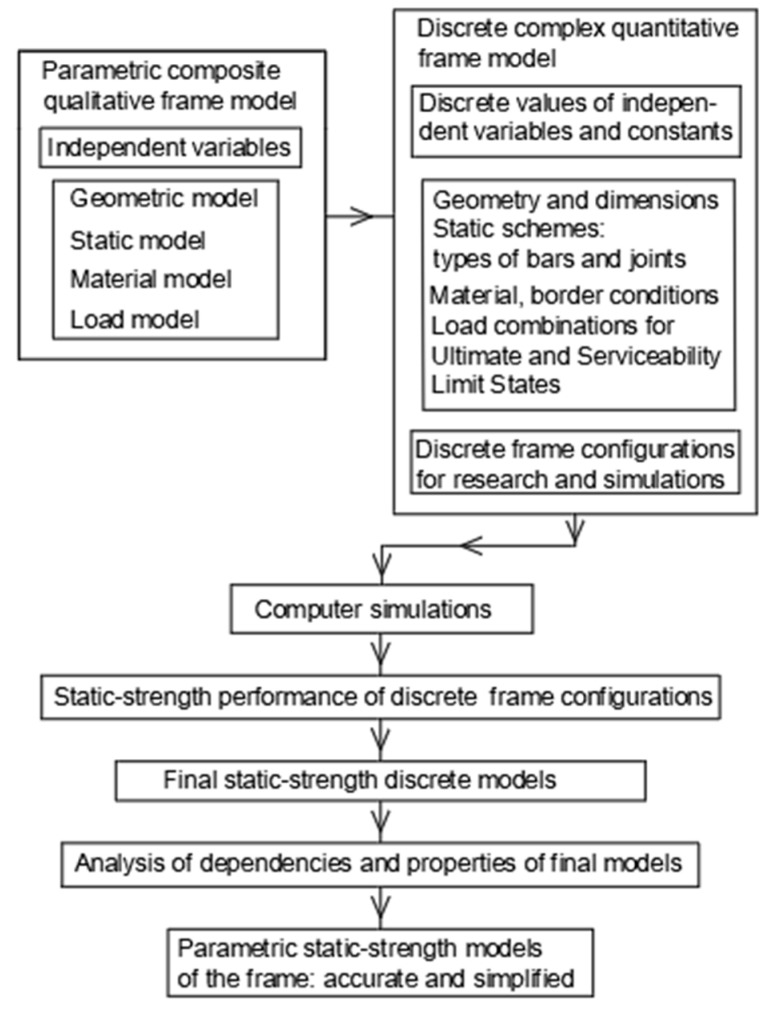
The methodology (the workflow) of the research.

The developed research concept, presented in Figure 4, in the form of a block diagram, requires defining subsequent discrete initial design models that will be used in computer simulations. In the first step of the research, the following subsequent simple models must be defined, geometric, static, material, physical and load, using independent variables (parameters) and constant coefficients. As a result of extending the definition to all the above-mentioned models, a complex parametric design model of the frame is created.

In the second step, small discrete values of independent variables were adopted to define the specific discrete frame models used in the subsequent simulations. In the third step, simulations of the stability and strength work of the constructed discrete models were performed. In the fourth step, based on the obtained results, the significance of the observed relationships was analyzed. Based on the above analysis, in the last step of the research, a resultant parametric static-strength frame model was developed.

**Figure 5:**
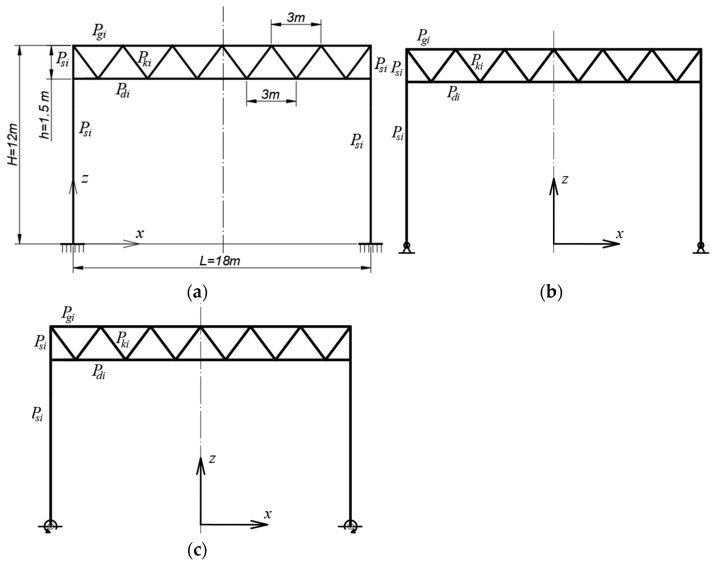
Three types of the examined frames: (**a**) a basic one with rigid column support, (**b**) a derivative frame with semi-rigid column support joints, (**c**) a derivative frame with articulated joints of column support (our own photos).

The geometric model of the frame was defined as a set of sections with dimensions as shown in Figure 5. Therefore, no geometric parameters were introduced. The static model of the frame was created from the above-mentioned geometric model by assuming that (1) each section is a model of one rod, (2) there are four types of elements, columns, lower chord, upper chord and diagonals, and (3) rods are rigidly connected at joints, except for column supporting joints, whose stiffness k (flexibility c) was assumed as a parameter—independent variable. Column members were designated as P_si_ (i = 1 to 4). Upper chord members were designated as P_gi_ (i = 5 to 10). Lower chord members were designated as P_di_ (i = 11 to 17). Diagonal members were designated as P_ki_ (i = 18 to 29).

Finally, it was assumed that the above-mentioned column joints are pinned, flexible or rigid. The Cfg1 configuration with rigid supporting joints, Figure 5a, was taken as a reference. For this configuration, an optimization was carried out. The optimizing conditions are described later in this section. The optimization concerned all types of elements belonging to Cfg1. The next tested derived configurations of Cfgi (i = 2 to 5) were characterized by similar cross-sections of elements as Cfg1. Mainly three types of Cfgi frame configurations with flexible joints were tested, in which the following stiffnesses were assumed: 50, 100 and 500 KNm/Deg; see Table 1.

The rotational stiffness of the column support joints, considered later in detail, is defined as the ratio of the bending moment occurring in the column support cross-section to the increase in the rotation angle of this cross-section caused by this moment [7,40]. The authors consider the joint initial stiffness. It is a sum of the stiffness of all individual components constituting the joint, mainly bolts and possibly gusset plates.

The material model is the extension of the geometric and static models. The material used is the S235 steel. The model also includes constraints on the yielding point of this steel, stability of the entire frame and its individual elements, displacements of the frame joints and deflections of the frame elements. The adopted mechanical properties are as follows: yield strength of the steel f_y_ = 235 MPa, modulus of elasticity E = 205 GPa, Kirchhoff modulus of shear deformation G = 80 MPa and specific weight of the steel ρ = 7800 kg/m^3^. The following optimizing constraints were adopted for the constructed models: (1) critical load factor f_cr_ ≥ 1.0, (2) maximum increase in the horizontal displacements of the frame joints Δx ≤ H/150, (3) maximum increase in the deflection of the girder Δz ≤ L/250.

The load model consists of the following instances: wind, snow, live loads and dead load of the frame bars. One type of wind load and one type of snow load characteristic for the lowland areas in Central Europe were assumed. In the calculations concerning the snow loads, ce = 1.0 and sk = 0.9 coefficients were assumed, which resulted in 0.72 kN/m^2^ [41]. In the calculations concerning the wind loads, v_D0_ = 19.8 m/s, q_b_ = 245 N/m^2^ and g_p_ = 0.593 kN/m^2^ [38] were assumed. In addition, a characteristic terrain height of z_ter_ = 300 m above sea level and a characteristic live load value of 0.3 kN/m^2^ were taken. Since the spacing of the transverse frame systems under consideration is 6 m, the values from Table 2 should be multiplied by 6 to obtain the load in kN/m for girders or columns.

For the above-mentioned individual instances of snow, wind, etc., standard load combinations were calculated [41,42]. For this purpose, the generally known Formulas (1a) and (1b) for the ULS limit state and (1c) for the SLS limit sate were used [43]. After substituting the appropriate coefficient values into these formulas, 12 ULS-type combinations and six SLS-type combinations were created in the manner described later in this section.

Kmbi = Σξ _j_ · γ_G,j_ · G_k,j_ + γ_Q,1_ · Q_k,1_ + Σγ_Q,i_ · Σψ_0,i_ · Q_k,i_ (1a)

Kmbi = Σγ_G,j_ · G_k,j_ + γ_Q,1_ · ψ_0,1_ · Q_k,1_ + Σγ_Q,i_ · ψ_0,1_ · Q_k,i_ (1b)

Kmbi = ΣG_k,j_ + Q_k,1_ + Σγ_Q,i_ · ψ_0,i_ · Q_k,i_ (1c)

where γ_G,j_ = 1.35 or 1.00, γ_Q,1_ = 1.5 or 0, γ_Q,i_ = 1.5 or 0, ξ = 0.85, ψ_0_ = 0.5 (for snow) or 0.6 (for wind).

In the case of the considered models and simulations, the following detailed loads and their combinations, acting on the building envelope, were assumed. It was assumed that in the first combination Komb1, snow is the leading load and (a) the load on the upper chord of the girder was calculated from:

K_Roof1 = 1.35 · 0.85 · G_R_ + 1.5 · S_1_ + 1.5 · 0.6 · (W_Rep,1_ + W_Ris,1_) (2a)

The column loads were calculated using formulas:

K_ScD1 = 1.35 · 0.85 · G_D_ + 1.5 · 0.6 · (W_Dep,1_ + W_Dis,1_) (2b)

K_ScE1 = 1.35 · 0.85 · G_D_ + 1.5 · 0.6 · (W_Eep,1_ + W_Eis,1_) (2c)

G_R_—the uniformly distributed load of the roof’s own weight; S_1_—the uniformly distributed snow load; W_Rep,1_—the uniformly distributed wind pressure on roof surfaces; W_Ris,1_—the suction pressure inside the building; G_D_—the distributed load of the walls’ own weight; W_Dep,1_—The uniformly distributed wind pressure on the windward wall; W_Dis,1_—the internal suction pressure acting on the windward wall; W_Eep,1_—the uniformly distributed wind suction acting on the leeward wall; W_Eis,1_—the internal suction pressure acting on the leeward wall.

It was assumed that in the case of the second combination Komb2, the leading load is the self-weight of the roof and walls and then (a) the load on the upper chord of the girder was calculated using:

K_Roof2 = 1.35 · G_R_ + 1.5 · 0.5 · S_1_ + 1.5 · 0.6 · (W_Rep,1_ + W_Ris,1_) (3a)

(b) while the column loads were calculated using formulas:

K_ScDE2 = 1.35 · G_D_ + 1.5 · 0.6 · (W_Dep,1_ + W_Dis,1_) (3b)

K_ScE2 = 1.35 · G_D_ + 1.5 · 0.6 · (W_Eep,1_ + W_Eis,1_) (3c)

In the case of the third load combination Komb3, the wind is the leading one and then (a) the load acting on the upper chord was calculated from the formula:

K_Roof3 = 1.35 · 0.85 · G_R_ + 1.5 · 0.5 · S_1_ + 1.5 · (W_Rep,1_ + W_Ris,1_) (4a)

(b) while the column loads were calculated using formulas:

K_ScDE3 = 1.35 · G_D_ + 1.5 · (W_Dep,1_ + W_Dis,1_) (4b)

K_ScE3 = 1.35 · G_D_ + 1.5 · (W_Eep,1_ + W_Eis,1_) (4c)

For the next three combinations Komb4, Komb5 and Komb6, formulas analogous to (2a)–(4c) were used, assuming that the upper chord of the girder is nonuniformly loaded with snow.

The Kombi combinations (i = 1 to 6) refer to the ultimate limit states of the frame. The following loads and their Kombi combinations (j = 7 to 9) presented in Formulas (5a)–(7c) were used for calculations related to the serviceability limit states of the frames under consideration.

Formulas (5a)–(5c) refer to combinations describing the possibility of roof lifting due to internal pressure and wind suction.

K_Roof7 = G_R_ + S_1_ + 0.6 · (W_Rep,1_ + W_Ris,1_) (5a)

K_ScDE7 = G_D_ + S_1_ + 0.6 · (W_Dep,1_ + W_Dis,1_) (5b)

K_ScE7 = G_D_ + S_1_ + 0.6 · (W_Eep,1_ + W_Eis,1_) (5c)

Equations (6a)–(6c) take into account the load combinations with a dominant share of snow.

K_Roof8 = G_R_ + 1.5 · S_1_ + W_Rep,1_ + W_Ris,1_ (6a)

K_ScDE8 = G_D_ + 1.5 · S_1_ + W_Dep,1_ + W_Dis,1_ (6b)

K_ScE8 = G_D_ + 1.5 · S_1_ + W_Eep,1_ + W_Eis,1_ (6c)

Equations (7a)–(7c) apply to combinations with asymmetric snow load.

K_Roof9 = G_R_ + S_1_ + 0.6 · (W_Rep,1_ + W_Ris,1_) (7a)

K_ScDE9 = G_D_ + S_1_ + 0.6 · (W_Dep,1_ + W_Dis,1_) (7b)

K_ScE9 = G_D_ + S_1_ + 0.6 · (W_Eep,1_ + W_Eis,1_) (7c)

The next nine combinations of Kombk (k = 10 to 18) are related to different wind directions acting on the building. In this case, the orientation of the frame girders in the building is parallel to the wind pressure direction, and the load from the side walls subjected to wind suction and internal pressure is transferred to the girder columns.

The Kombi combinations (i = 10 to 15) refer to the ultimate limit states (ULSs) of the frame analogously to the Kombi combinations (i = 1 to 6), with the difference that in these cases Equations (8a)–(8c) are used to calculate the loads acting on both columns of the frames. Combinations for asymmetric snow loads are not given here because of the analogy to those for the symmetrical snow loads.

K_ScB10 = 1.35 · 0.85 · G_D_ + 1.5 · S_1_ + 1.5 · 0.6 · (W_Bep,2_ + W_Bis,2_) (8a)

K_ScB11 = 1.35 · G_D_ + 1.5 · 0.5 · S_1_ + 1.5 · 0.6 · (W_Bep,2_ + W_Bis,2_) (8b)

K_ScB12 = 1.35 · 0.85 · G_D_ + 0.5 · 1.5 · S_1_ + 1.5 · (W_Bep,2_ + W_Bis,2_) (8c)

In turn, for calculations related to the serviceability limit states (SLSs) of the considered frames, the Kombi load combinations (j = 16 to 18), presented in Formulas (9a)–(9c), were used.

K_ScB17 = G_D_ + 1.5 · (W_Bep,2_ + W_Bis,2_) (9a)

K_ScB17 = G_D_ + S_1_ + 0.6 · (W_Bep,2_ + W_Bis,2_) (9b)

K_ScB18 = G_D_ + 0.5 · S_1_ + W_Bep,2_ + W_Bis,2_ (9c)

The values of live load, wind, snow and internal pressure acting on the roof and individual walls of the building were calculated for each of the above-mentioned load combinations and presented in Table 3. The dead weight of the frame elements was calculated by the ARSA computer program used in the simulations [44].

**Figure 6:**
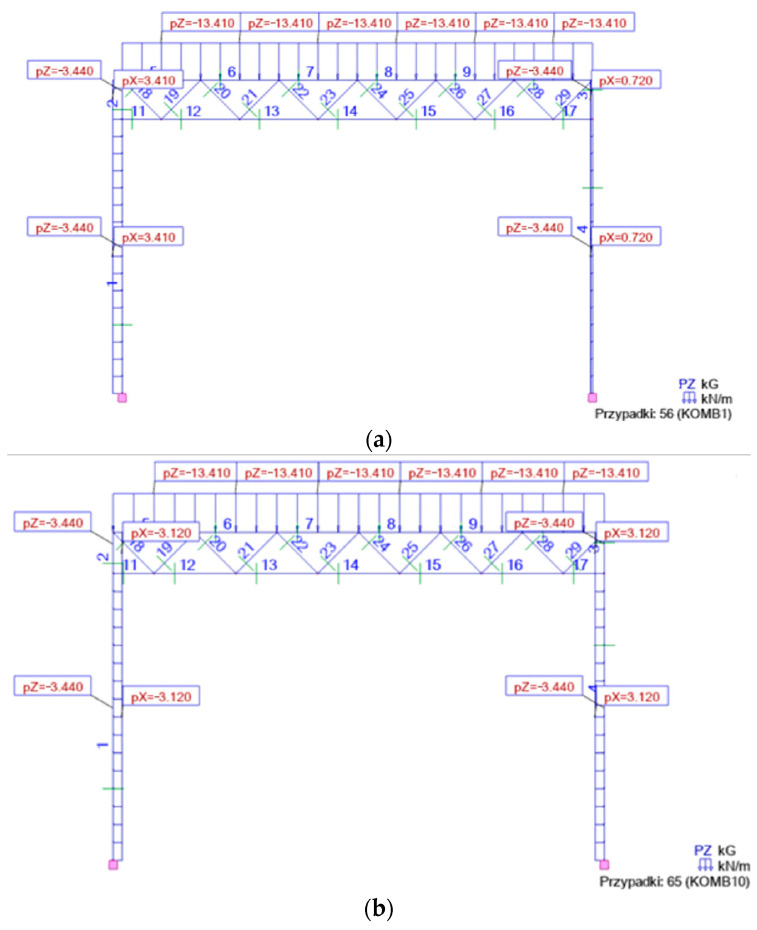
Ultimate limit state: (**a**) Komb1 with asymmetric wind load on gable walls and symmetrical snow load on roof; (**b**) Komb2 with symmetrical wind load on side walls and symmetrical snow load on roof.

**Figure 7:**
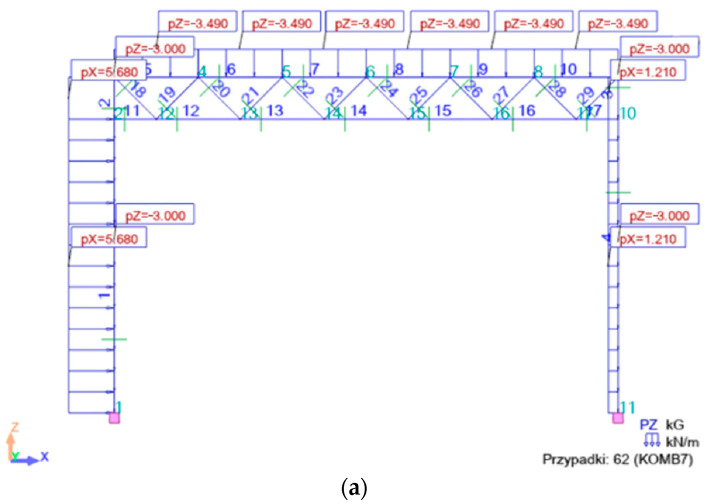

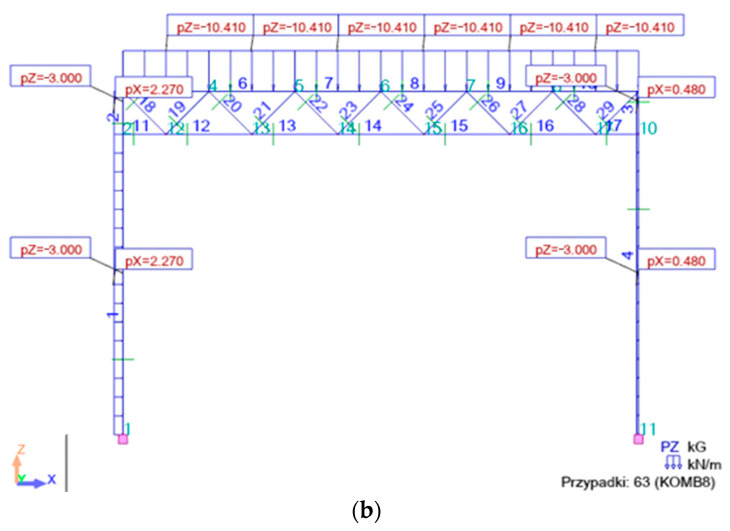
Serviceability limit state: (**a**) Komb7 with corrected wind load on gables and roof and full roof snow load; (**b**) Komb16 with full wind load on gables and roof and corrected roof snow load.

Examples of two combinations, Kmb1 and Kmb10, meeting the ULS requirements are presented in Figure 6; examples of two combinations, Kmb8 and Kmb9, meeting the SLS requirements are presented in Figure 7.

The parametric computational model of a flat frame, developed for simulations, is a sum of the initial models defined above: geometric, static, material and load. Based on the results of computer simulations, related to discrete values and models obtained during the subsequently performed simulations, a relatively complex parametric static-strength model of the frame was created [33]. This model is defined mainly by dependent variables, i.e., parameters dependent on the only independent variable, i.e., the rotational stiffness of the column support joints. The nonlinear incremental finite element method [45,46] was used to calculate discrete values of dependent variables describing the final static-strength frame model.

## 5. Results

The results of the strength and stability simulation of the first tested group of frame configurations Cfgi (i = 1 to 5) are presented in Table 4. Analogous elements of each of these configurations are characterized by identical cross-sections, which means that the cross-sections of the Cfgi configurations (i = 2 to 5) were intentionally not optimized due to the previously presented optimizing conditions and simulation goals. The Cfg1 configuration with fixed columns was taken as the reference and the cross-sections of all its elements were optimized, while maintaining the conditions given in the methodology section.

In the case of the optimized reference configuration Cfg1, the non-exceedance of the permissible stresses for all of its elements turned out to be the decisive condition. The frame maintained sufficient stability and stiffness under the load, so the permissible displacements of its joints and deflections of its elements as well as the critical load factor were not exceeded. In the case of the subsequent derivative configurations Cfgi (i = 2 to 5) with flexible column supporting joints, the following are noticeable: (1) an increase in stresses above the permissible levels, primarily in the columns, as well as in the upper chord and diagonals, (2) a radical decrease in the stiffness of the frame in the horizontal direction transverse to the height of the columns.

The results of the static and strength simulation of the second group of Copi frame configurations (i = 2 to 5) are presented in Table 5. Since the reference configuration Cfg1 was optimized in the previous stage, it was assumed that it is the basic Copi1 configuration of the second optimized group Copi. The optimization was performed for each type of element of each subsequently simulated Copi frame. As a result of the optimization process, configurations characterized by similar stiffness and stress of their analogous elements were obtained, except for the columns.

There exists a significant trend to increase the cross-sectional area of the columns for the successive configurations meeting the optimization conditions. Because of the continuous nature of the load transferred from the corrugated sheets perpendicularly to the upper chord bars of the girder, it was decided to provide the section modules of the cross-sections of the extremely stressed bars.

## 6. Analysis

### 6.1. Relationships Characteristic for the First Type Cfgi of the Frame Configurations

**Figure 8:**
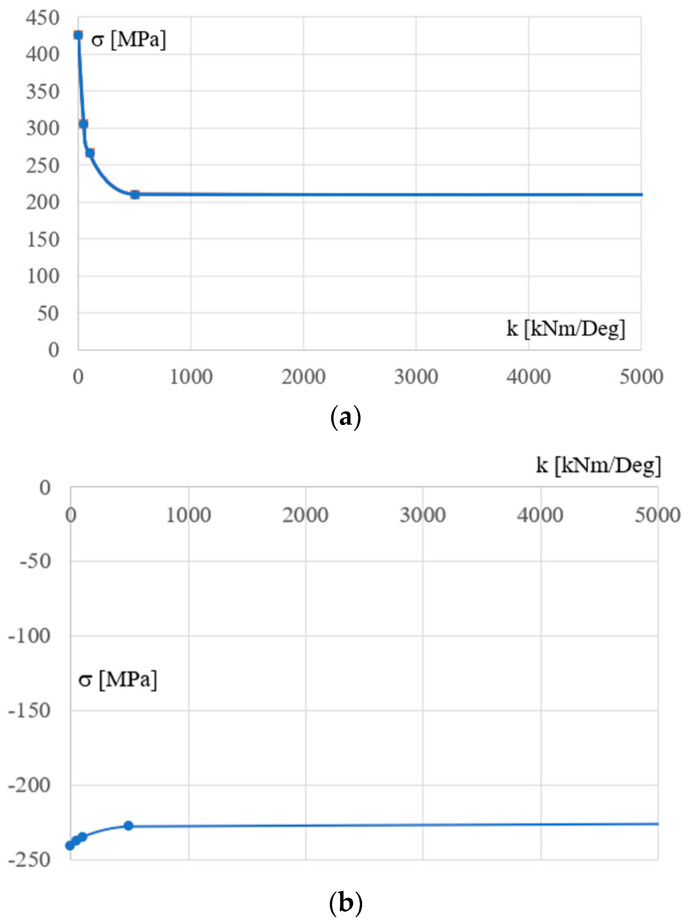
The dependence between the k stiffness of the column’s support and the *σ* maximum stresses occurring in (**a**) the columns and (**b**) the bottom chords of the analyzed frames.

Based on the values given in the tables presented earlier, the following diagrams were developed to illustrate the observed relationships between the calculation (design) model and the resultant model. The relationships obtained for the Cfgi configurations (i = 1 to 5) are presented in Figure 8a. The line from this diagram represents a nonlinear relationship between the k rotational stiffness of the column’s support and the maximum stresses *σ* occurring in the columns. The relationship is strongly nonlinear. A decrease in the stiffness of the support joints 400 to 0 kNm/Deg causes a significant change, i.e., an increase, in the stresses in the column by up to approx. 100%. However, in the case of the lower chord, Figure 8b, the analogous relationship is weakly nonlinear. An increase in the stiffness of the support joints from zero to very high value causes a relatively small decrease in the absolute values of the stresses of the girder lower chord by up to 15%. In the case of other elements, the changes are insignificant.

**Figure 9:**
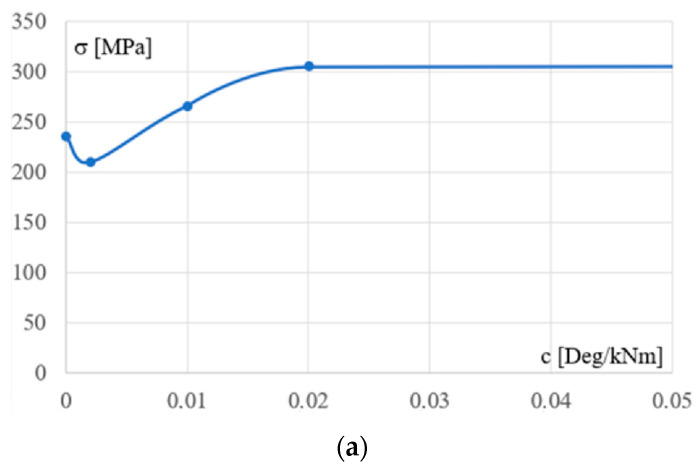

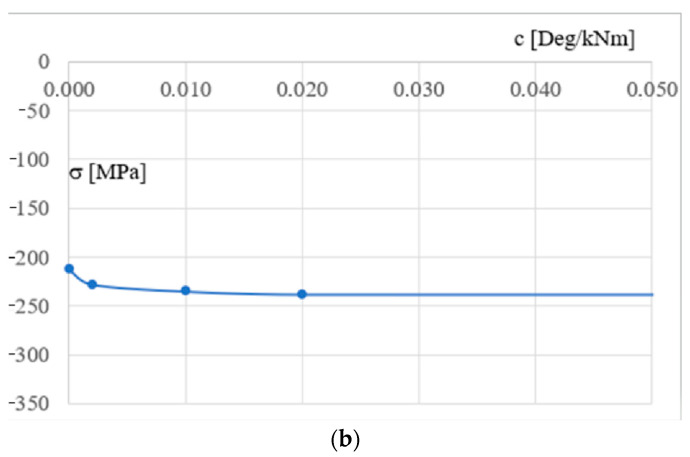
The dependence between the flexibility c of the column’s support and the maximum stresses occurring in (**a**) the column and (**b**) the girder bottom chord.

Figure 9 shows the relationship between the flexibility c of the column support and the maximum stresses occurring in the column, Figure 9a, and the bottom chord, Figure 9b, respectively. In the case of columns, this relationship is strongly nonlinear with a local extremum. In the case of the girder bottom chord, the relationship is weakly nonlinear.

**Figure 10:**
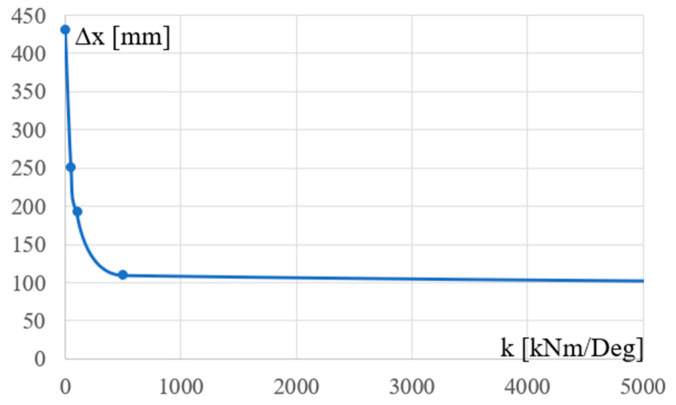
The dependence between the stiffness k of the column’s support and the maximum horizontal displacement of the column’s top joints.

In addition to the load combinations defined for ULSs, the load combinations characteristic for SLSs were considered. Figure 10 presents the relationship between the stiffness k of the column’s support and the maximum horizontal displacement of the column top joints calculated for the Cfgi configurations unoptimized due to the optimizing conditions set for Cfg1 and presented earlier. This relationship is strongly nonlinear.

**Figure 11:**
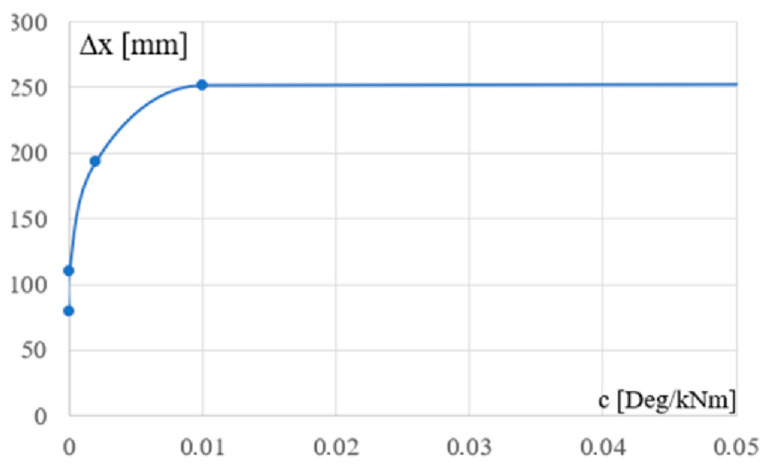
The dependence between the c flexibility of the column’s support and the maximum horizontal displacement of the column’s top of the analyzed frames.

The curve from Figure 11 shows the relationship between the flexibility c of the column support and the maximum horizontal displacement of the common joint of the column and the bottom chord. This relationship is, of course, also strongly nonlinear for the Cfgi (i = 2 to 5) configurations.

### 6.2. Relations Characteristic for the Copi Frame Configurations Optimized

As a result of the optimization process of the cross-sections of all elements of Copi, the diagrams presented in the next part of this subsection were obtained. The number of diagrams was limited to the cases of column operation, since only in this case the obtained dependencies are significant.

**Figure 12:**
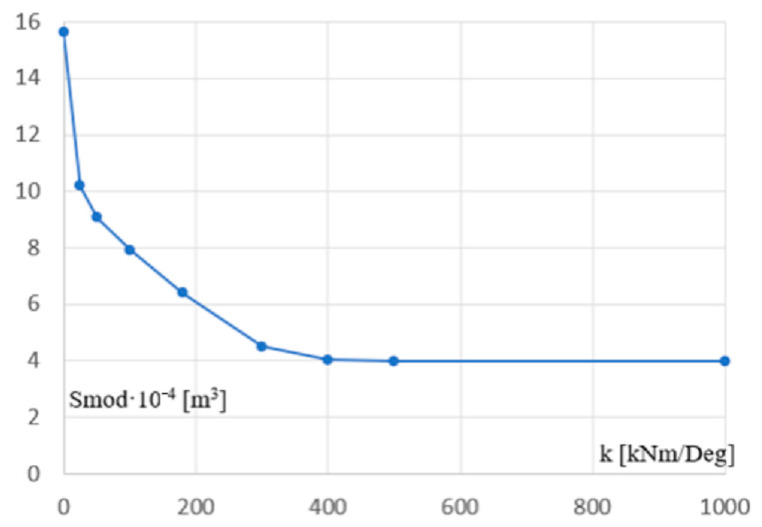
The dependence between the stiffness k of the column’s support and the section module Smod of the column’s cross-section.

Figure 12 presents the relationship between the stiffness k of the support and the cross-section module of the column. This relationship is strongly nonlinear and very difficult to model using an analytical description, for example, a single equation. Therefore, to obtain the appropriate accuracy of the resulting model description, it was decided to approximate the line from Figure 12 with a segment line. The obtained results regarding the analytical description of this line are presented in the next subsection.

The simulation results and analysis of the diagram presented in Figure 12 indicate a fundamental tendency consisting in a strong decrease in the size of the column’s cross-sections, obtained during the optimizing process of the static-strength performance of the frames, caused by a slight increase in the rotational stiffness of the supporting joints of these columns in the range from 0 to 30 kNm/Deg. This tendency justifies the rationality of making flexible connections, e.g., bolted ones, instead of shaping hinges.

In turn, increasing the stiffness of the above joints, for example to a value of over 300 kNm/Deg, does not result in satisfactory reduction of the above-mentioned cross-section size obtained during the optimizing process. This trend indicates the inefficiency and inappropriateness of using rigid connections in the most commonly used typical bar frames and typical load and environmental conditions.

On the other hand, the change in stiffness in the range from 30 to 300 kNm/Deg causes a relatively slow decrease in the size of the column cross-sections, obtained during the optimizing process, caused by the increase in the stiffness of the supporting joints. This tendency is depicted by the not too large angle of inclination of the middle section belonging to the line from Figure 12. In this case, the selection of the appropriate joint stiffness should be made as a result of the analysis of the frame construction costs and the contractor’s technological possibilities. However, the description of such actions goes beyond the scope of this article.

**Figure 13:**
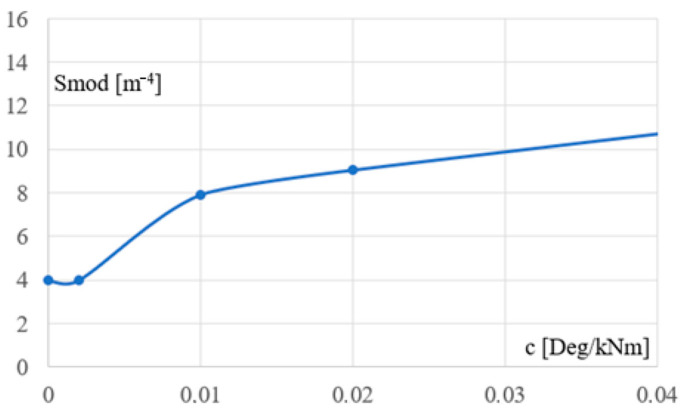
The dependence between the flexibility c of the column’s support and the section modulus of the column’s cross-section.

Figure 13 presents the relationship between the flexibility c of the column support and the cross-sectional section module of the column. This relationship is also strongly nonlinear. This line could also be approximated by a line composed of a few straight segments. However, it is not a part of the resultant model, so such activities go beyond the scope of this article.

**Figure 14:**
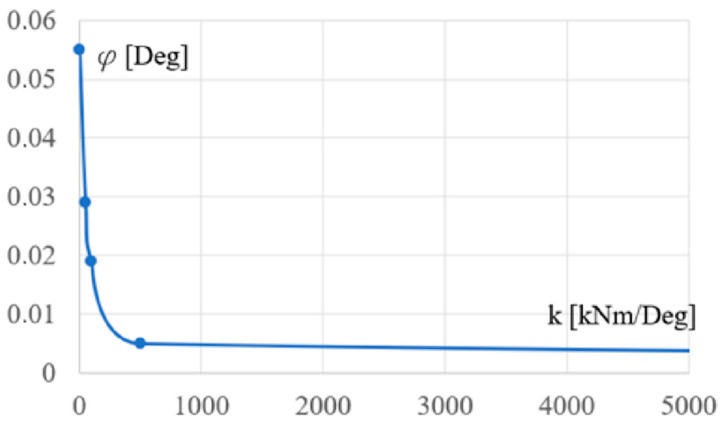
The dependence between the stiffness k and the rotation angle f of the column’s support.

Figure 14 presents the relationship between the stiffness k of the column support and the rotation angle of the column. This relationship is also strongly nonlinear. It is not a part of the resultant model, so such activities go beyond the scope of this article.

### 6.3. Parametric Model of the Column Support Joint

The relationship between the supporting joint stiffness k and the section module Smod of columns, presented in the previous section, can be modelled as a function Smod = f(k) that determines the rational static-strength performance of the columns and entire frame. It is important to note the non-linear nature of this dependence especially in the range of 50 to 500 kNm/Deg.

**Figure 15:**
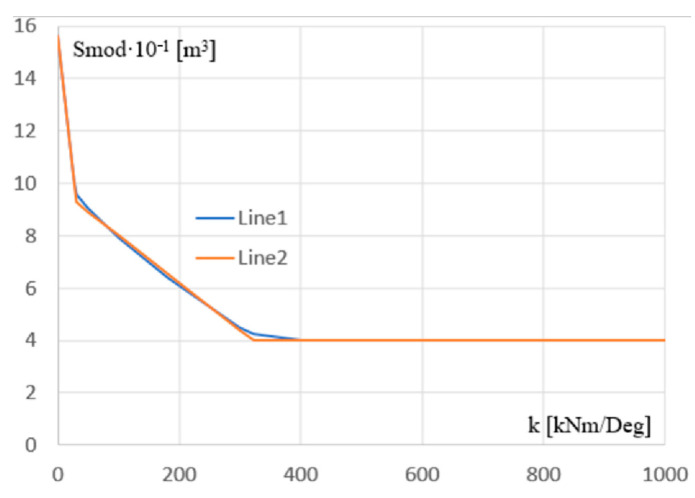
Two lines presenting the dependence between the k stiffness of the column’s support and the Smod section module of the column’s cross-section: (a) Line 1 obtained based on the simulated models and their discrete characteristics; (b) Line 2—the three-segment line with a given analytical equation approximating Line 1.

The line from Figure 12, represented by Line 1 in Figure 15, was finally approximated by the optimized line Line 2 composed of three segments. These segments were determined based on three straight lines pr1, pr2, and pr3, with Equation (10) and two points Q1 and Q2 of intersection of these lines.

(10) $$y=a_{i}\cdot x+b_{i}$$

where i = 1 to 3.

The coefficients $a_{i}$ and $b_{i}\;$ of the $p_{i}$ straight lines and the coordinates of their intersecting points are given in Table 6. The coordinates of each of these points are the stiffness k of the support joint and the section module Smod of the column. The defined segment line is the main part of the searched parametric model determining the optimal performance of the examined frames.

**Figure 16:**
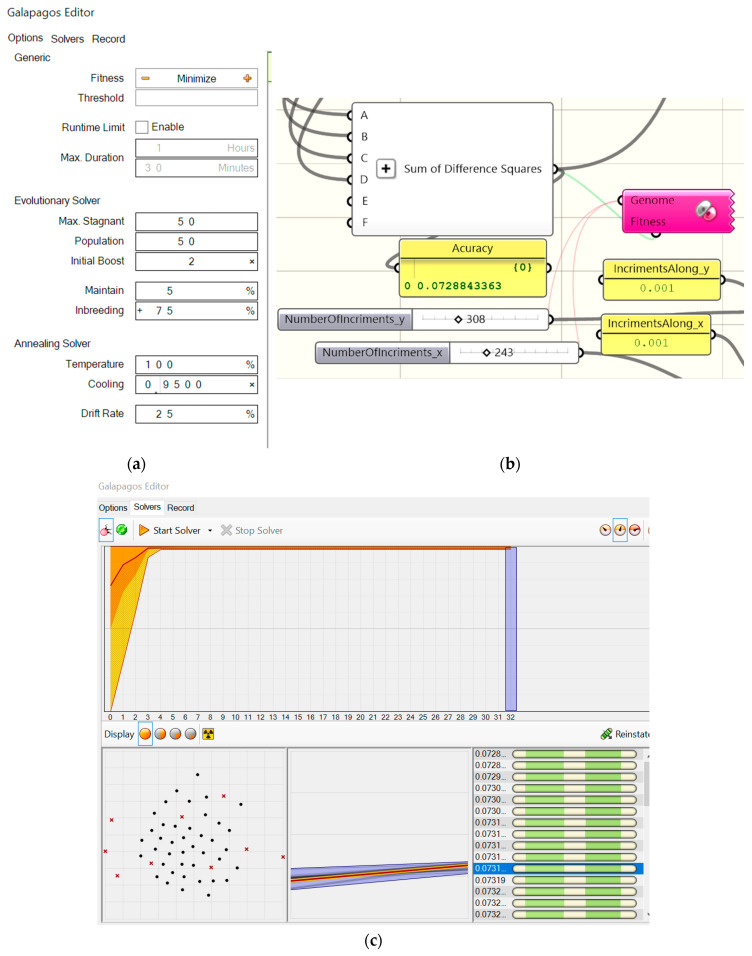
The values of the configuration parameters of the Galapagos optimization program employed (**a**), the invented objects developed in the Rhino/Grasshopper program, including the accuracy container, carrying out the optimization process (**b**), the interface displaying the current results and progress of the performed optimization process (**c**).

Figure 16a–c presents (1) the adopted values of the configuration parameters of the Galapagos optimization program, Figure 16a, (2) the invented objects implementing the elaborated procedures of the optimization process developed in the Rhino/Grasshopper program [47], Figure 16b, (3) the achieved accuracy of the optimization process performed, Figure 16b, (4) the interface displaying the current results and progress in the optimizing activities of the Galapagos application, Figure 16c. The obtained accuracy of 0.072884 refers to the units used for the x and y axes of the diagram from Figure 15 and the nine points represented by the dots in Figure 12.

The genetic algorithm was used to develop the diagram, i.e., optimize the position and inclination of the lines on the diagram from Figure 15, based on the achieved results. However, it was not used to optimize the simulation results. The function of this algorithm is going to be expected in future studies, where it is planned to take into account a much larger number of independent variables. The least squares method was implemented for this optimization algorithm.

## 7. Discussion

The various stiffnesses of the support joints of single-branch columns, considered as pinned, semi-rigid or rigid, lead to the following: (1) significant differentiation of the effort of columns, including the stress differentiation at the level of approx. 100%, (2) relatively small differentiation of the strength of the lower chords of the examined frame girders, including the stresses differing at the level of approximately 8%. In the case of the remaining elements of the simulated frames, the changes in the stresses are negligible. The decrease in the column joint stiffness causes a non-linear decrease in stresses, especially when reducing stiffness in the range from 300 to 0 kNm/Deg. An increase in the stiffness of the support joints to values above 300 kNm does not cause a noticeable change in the column stress state.

The decrease in the stiffness of the column support joints also causes (1) a very significant increase in the horizontal displacements of the top frame joints, including the column and girder connections, exceeding the permissible standard values Δx_max_, (2) an insignificant increase in the girder deflection, not exceeding the standard limits Δz_max_, (3) a significant increase in the rotation of the column support end, (4) relatively small changes in stability within the safe range of frame operation.

The strongest limitation in shaping the single-branch column frames relates to the horizontal displacements of joints to the values allowed by the standard and amounting to Δ*x* = H/150 = 12,000/150 = 80 mm. This condition did not allow for the use of the full load-bearing capacity of columns with semi-rigid support joints. The analysis carried out indicates that the possibility of using the full load-bearing column capacity requires changing the types of columns or introducing additional frame stiffeners in the horizontal direction.

For the above reasons, the optimization of the cross-sections of all elements was carried out for each frame configuration with different types of supporting joints in the second step of the performed research. The analysis of the obtained results led to the fairly and accurate description of the column’s joint performance and the influence of the joints on the static-strength work of the columns and entire frame. Next, the analysis made it possible to define the simplified parametric model of rational transverse frame structural systems working effectively under specific external loads. The expected extension of this model with further geometric, physical and load parameters will consist in simulations and analysis analogous to those presented in this paper.

The considered issues concerned the location of buildings in temperate climate zones characterized by medium wind and snow loads. The spacing, length and height of the frame configurations under consideration were unchanged and amounted to 18 and 12 m. In addition, the girder height was 1.5 m. The analysis carried out showed the significance of the influence of the column support stiffness on the mechanical properties of the simulated frames. This significance changes in a non-linear manner depending on the change in the rotational stiffness of the column support joints. New research is also planned related to the influence of the stiffness of the column connections with the girder chords. The influence seems to be also significant if we analyze, among other things, the research results presented in the article.

Thus, it is advisable to increase the number of independent variables of the parametric design frame model shaped. To increase the universality of the model, it is necessary to increase the number of independent variables, which will cause several changes in the static-strength properties of the frames. The obtained and presented results indicate a very high probability of the significant influence of the above-mentioned factors. Such trends determine the direction of the new future research and allow for the high probability of expecting satisfactory results.

## 8. Conclusions

A new single-parameter mechanical model of a flat bar frame with a strictly defined static scheme was developed. The parameter is stiffness (flexibility) of column support joint. The examined frames are composed of lattice girders and single branch columns. The analyzed influence of the support joint stiffness on the geometric properties of the optimized cross-sections of the bars of individual frame elements turned out to be significant. The optimizing conditions used are as follows: (1) general stability defined by the critical load factor, (2) stresses in the range up to the yield point, (3) displacements of joints, (4) deflections of frame elements with consideration of the ultimate and serviceability limit states, (5) loads characteristic of a moderate climate.

The increase in the rotation stiffness of the joints from zero through intermediate values up to complete restraint necessitates changes in the cross-section sizes of the optimized individual elements in the following ranges. Reducing the support joint stiffness from 300 to 100 kNm/Deg requires an increase in the column section module from 4.5·10^−4^ to 7.9·10^−4^ m^3^, i.e., by approx. 90%. However, a further reduction of the stiffness from 100 to 0 kNm/Deg makes it necessary to increase the column section module from 7.9 to at least 15.6·10^−4^ m^3^. In the case of the lower chord, the decrease in the support joint stiffness from 400 to 0 kNm/Ded requires a change in the cross-sectional area of its bars from 8.78·10^−2^ to 13.77·10^−2^ cm^2^, which is about 55%. In the case of the upper chord and diagonals, there are almost no changes in the cross-sections.

Finally, a detailed description of the nonlinear effect of the column support joint stiffness on the optimized cross-sections of all frame elements was defined with the help of the presented analytical formulas and graphical diagrams. Since the accuracy of a number of analytical formulas describing the observed nonlinear relationships is low, it was necessary to determine the relatively complex descriptions by means of the three-segment line. Such a procedure required the use of the least squares method and proprietary procedures implemented in the Rhino/Grasshopper program, as well as the generally known genetic optimizing algorithms.

To build general universal models, a number of sets of discrete values obtained during tests for subsequently simulated frame configurations with different support joint stiffnesses were used. Based on these discrete data, the relationships between the independent variable defining the stiffness and the dependent variables, such as section modules or cross-section areas, defining the resultant parametric mechanical model of the frame were examined. The developed parametric model can be modified by adding subsequent new parameters in the manner presented for the column support joint stiffness. Thus, the presented algorithm allows for achieving greater universality of the created models by introducing a number of variables controlling the geometric and physical properties of the designed frames and their loads.

## Figures and Tables

**Table 1:** The tested frame configurations with different types of supporting joints.

Configuration	Cfg1	Cfg2	Cfg3	Cfg4	Cfg4
Type of the joints	Rigid	Semi rigid	Semi rigid	Semi rigid	Articulated
Stiffness k (kNm/Deg)	5000	500	100	50	0
Flexibility c (Deg/kNm)	0.000	0.01	0.02	100	10,000
D_op_cl_/d_op_cl_ (mm)	406.4/6.3	406.4/6.3	406.4/6.3	406.4/6.3	406.4/6.3
D_op_tp_/d_op_tp_ (mm)	168.3/4.5	168.3/4.5	168.3/4.5	168.3/4.5	168.3/4.5
D_op_bt_/d_op_bt_ (mm) D_op_dg_/d_op_dg_ (mm)	114.3/5.0 70/3.6	114.3/5.0 70/3.6	114.3/5.0 70/3.6	114.3/5.0 70/3.6	114.3/5.0 70/3.6

**Table 2:** The load characteristics of the simulated models.

Girder		Column	
Coefficient	Value	Coefficient	Value
GD (kN/m^2^)	1.04	GSc (kN/m^2^)	0.5
S1 (kN/m^2^)	0.72	WDep + Wis (kN/m^2^)	0.631
S2 (kN/m^2^)	0.36	WEep + Wis (kN/m^2^)	0.134
W_ep_ + W_is_ (kN/m^2^)	−0.042	WBep + Wis (kN/m^2^)	0.631
W_es_ + W_ip_ (kN/m^2^)	−0.31		

**Table 3:** The values of load acting on the roof and walls of the building for each of the load combinations considered.

	Roof (kN/m^2^)	Winward Wall (kN/m^2^)	Leeward Wall (kN/m^2^)	Side Walls (kN/m^2^)	Wall’s Weight (kN/m^2^)
Komb1	2.24	0.57	0.12	-	0.5737
Komb2	1.91	0.57	0.12	-	0.675
Komb3	1.67	0.95	0.20	-	0.5737
Komb4	1.70	0.57	0.12	-	0.5737
Komb5	1.64	0.57	0.12	-	0.675
Komb6	1.40	0.95	0.20	-	0.5737
Komb7	0.58	0.95	0.20	-	0.5
Komb8	1.73	0.38	0.08	-	0.5
Komb9	1.36	0.63	0.13	-	0.5
Komb10	2.24	-	-	0.52	0.5737
Komb11	1.91	-	-	0.52	0.675
Komb12	1.67	-	-	0.87	0.5737
Komb13	1.70	-	-	0.52	0.5737
Komb14	1.64	-	-	0.52	0.675
Komb15	1.40	-	-	0.87	0.5737
Komb16	0.58	-	-	0.87	0.5
Komb17	1.73	-	-	0.35	0.5
Komb18	1.36	-	-	0.58	0.5

**Table 4:** Stress and stability characteristics of the tested frame configurations characterized by constant cross-sections of analogous elements, and without cross-section optimization.

	Roof (kN/m^2^)	Winward Wall (kN/m^2^)	Leeward Wall (kN/m^2^)	Side Walls (kN/m^2^)	Wall’s Weight (kN/m^2^)
Configuration	Cfg1	Cfg2	Cfg3	Cfg4	Cfg5
σ_max_cl_/σ_min_cl_	236/−218	230/−201	289/−260	333/−303	482/−448
σ_max_tp_/σ_min_tp_	233/−123	234/−126	237/−131	239/−134	238/−154
σ_max_bt_/σ_min_bt_	179/−226	193/−231	231/−238	259/−241	335/−241
σ_max_dg_/σ_min_dg_	232/−236	232/−236	239/−246	245/−254	263/−277
Δx (mm)	38	39	40	40	40
Δz (mm)	58	79	136	170	316
Δf (Deg)	0.007	0.007	0.014	0.021	0.045
k (kNm/°)	Rigid	500	100	50	0

**Table 5:** Geometric and mechanical characteristics of the tested frame configurations with different cross-sections of analogous elements of the optimized frames.

Configuration	Cop2	Cop3	Cop4	Cop5	Cop6	Cop7	Cop8
D_op_cl_/d_op_cl_	406/6.3	457/5.6	457/8.0	508/8.0	610/6.3	610/7.1	711/8.0
D_op_tp_/d_op_tp_	168.3/4.5	168.3/4.5	168.3/4.5	168.3/4.5	168.3/4.5	168.3/4.5	168.3/4.5
D_op_bt_/d_op_bt_	114.3/5	114.3/5.0	114.3/5	114.3/4.5	114.3/5.0	114.3/5.6	177.8/5.0
D_op_dg_/d_op_dg_	70/3.6	70/3.6	70/4.0	70/4.0	70/4.0	70/4.0	70/4.0
S_mod_cl_ (cm^3^)	399,205	450,910	639,090	791,781	906,414	1,019,502	1,561,537
S_mod_tp_ (cm^3^)	1174	1174	1174	1174	1174	1174	1174
S_mod_bt_ (cm^3^)	878	878	878	792	878	981	1377
S_mod_dg_ (cm^3^)	386	386	427	427	427	427	427
k (kNm/Deg)	500	300	180	100	50	25	0

**Table 6:** The coefficients of three straight lines taking part in the approximation process and the coordinates of the points of intersection of these lines.

Coefficient	$a_{i}$	$b_{i}$
Line pr1	−13.09	15.62
Line pr2	−1.802	9.808
Line pr3	0.000	4.000
Coefficient	k (kNm/Deg)	Smod (m^3^)
Point Q1	29.25	9.281
Point Q2	322.3	4.000

## Data Availability

The original contributions presented in this study are included in the article. Further inquiries can be directed to the corresponding author.

## Nomenclature

Cfgi	the unoptimized frame configuration
Copi	the optimized frame configuration
k	the stiffness of the column supporting joints
c	the flexibility of the column supporting joints
P_gi_	the upper chord of a girder
P_di_	the lower chord of a girder
P_ki_	the diagonal of a girder
P_si_	the column
D_op_cl_, d_op_cl_	the outer diameter/thickness of the column sections
D_op_tp_/d_op_tp_	the outer diameter/thickness of the top chord sections
D_op_bt_/d_op_bt_	the outer diameter/thickness of the bottom chord sections
D_op_dg_/d_op_dg_	the outer diameter/thickness of the diagonal sections
S_mod_cl_	the section module of the column cross-sections
S_mod_tp_	the section module of the top chord cross-sections
S_mod_bt_	the section module of the bottom chord cross-sections
S_mod_dg_	the section module of the diagonals cross-sections
*σ_c_*	the compressive stresses
*σ_t_*	the tensile stresses
f*_cr_*	the critical load factor
Δx_max_	the maximum displacement in the *x*-axis direction
Δz_max_	the maximum deflection in the *z*-axis direction
[x, y, z]	the orthogonal coordinate system
(x, z)	one of three principal planes of [x,y,z]
